# Worry as a window into the lives of people who use injection drugs: a factor analysis approach

**DOI:** 10.1186/1477-7517-6-20

**Published:** 2009-07-29

**Authors:** Heidi Exner, Erin K Gibson, Ryan Stone, Jennifer Lindquist, Laura Cowen, Eric A Roth

**Affiliations:** 1 AIDS Vancouver Island, 1601 Blanshard Avenue, Victoria, British Columbia, V8W 2J5, Canada; 2 Department of Mathematics and Statistics, PO BOX 3060 STN CSC, Victoria, British Columbia, V8W 3R4, Canada; 3 Department of Anthropology, University of Victoria, PO Box 3050, STN CSC, Victoria, British Columbia, V8W 3P5, Canada

## Abstract

**Background:**

The concept of risk dominates the HIV/AIDS literature pertaining to People Who Use Injection Drugs (PWUID). In contrast the associated concept of worry is infrequently applied, even though it can produce important perspectives of PWUID's lives. This study asked a sample (n = 105) of PWUID enrolled in a Victoria, British Columbia needle exchange program to evaluate their degree of worry about fourteen factors they may encounter in their daily lives.

**Methods:**

Exploratory factor analysis was used to analyze their responses.

**Results:**

Factor analysis delineated three factors: 1) overall personal security, 2) injection drug use-specific risks including overdosing and vein collapse and, 3) contracting infectious diseases associated with injection drug use (e.g. HIV/AIDS and hepatitis C).

**Conclusion:**

PWUID in this study not only worry about HIV/AIDS but also about stressful factors in their daily life which have been linked to both increased HIV/AIDS risk behaviour and decreased anti-retroviral treatment adherence. The importance PWUID give to this broad range of worry/concerns emphasizes the need to place HIV/AIDS intervention, education, and treatment programs within a broader harm-reduction framework that incorporates their perspectives on both worry and risk.

## Background

Injection drug use is a driving force in historic HIV epidemics in North America and emerging epidemics in Asia and Eastern Europe [1], and is the world-wide leading cause of hepatitis C infection [2]. As a result the public health and epidemiological literature on People Who Use Injection Drugs (PWUID) [3] is dominated by the concept of "risk", associated largely with the sharing of injection drug equipment. Applications of the concept of risk to PWUID originated with seminal studies on disease transmission parameters [4,5], evolved to consider risk networks [6,7], and presently focus on risk environments [8] and the structural production of risk [9].

In contrast to this long-standing concern with the concept of risk there has been relatively little development of a related concept, that of worry, in the HIV/AIDS literature. A notable exception is Smith and Watkins' [10,11] substitution of "worry" about contracting HIV/AIDS in place of "risk of HIV/AIDS". In doing so they argue that worry is an important concept since, "worry is universally experienced and more emotionally based than perceived risk, respondents may have less difficultly understanding the concept of worry and articulating their levels of worry than describing their perceived risk" [10:72]. To this we add that consideration of what people worry about can help identify what individuals and/or groups perceive as actually constituting risks, since risk is now assumed to be socially constructed [9]. From this perspective we can define worry as the recognition of risk. As such consideration of what people worry about has the potential to provide important "windows" into their lives by identifying the risks and challenges they face. This is exemplified by Busza's [12] study of worry patterns for Vietnamese sex workers in Cambodia, which revealed worry about HIV/AIDS nested within broader frameworks encompassing the need to provide funds for extended families in Vietnam, issues of police harassment, and demanding brothel owners.

For PWUID the importance of considering broader factors of concern was highlighted by Mizuno et al. [13] who asked HIV-seropositive PWUID to rank order their "life priorities" from a list of seven items including HIV, housing, having money, food, being able to work, childcare, and safety from violence. In their sample of 161 individuals only 37% ranked HIV as a top priority, while nearly half ranked HIV as their fourth priority or lower. Similarly Brogly *et al*. [14] asked Montreal PWUID to choose five cards representing what they perceived as the most important factors in their quality of life from a group of seventeen cards including HIV/AIDS treatment, drug treatment, health, being useful, education, feeling good about yourself, independence and free choice, spirituality, friends, family, partnership, sex, housing, money, resources leisure activities, and drugs. From this list the most frequently chosen factors were housing, health, money, spirituality, family, and feeling good about yourself. Only 13% of participants included HIV/AIDS treatment as an important factor, and this factor ranked a lowly twelfth out of the possible seventeen items.

This recognition of worries in addition to HIV/AIDS infection or treatment is important because both ethnographic studies [15] and health surveys [16] report on the chaotic nature of PWUID's lives, with homelessness, stigma, and lack of resources associated with high-risk behaviours ranging from sharing injection drug paraphernalia to survival sex, while simultaneously acting as barriers to HIV/AIDS treatment [17]. In the present paper we analyze responses to a survey questionnaire to delineate worry patterns for PWUID enrolled in a long-established needle exchange program in Victoria, British Columbia in order to gain a broader understanding of what they identify as risk and worry about in their everyday lives.

## Methods

Data for this study were generated by a survey of AIDS Vancouver Island's Street Outreach Services (AVI-SOS) Needle Exchange Program clientele. Conducted in April-May/2008 the survey represented a collaborative research project between members of AIDS Vancouver Island and the University of Victoria designed to address the issue of continued injection drug equipment sharing among needle exchange clientele [18]. AIDS Vancouver Island has a long-running Street Outreach Services (AVI-SOS) Needle Exchange Program. Established in the early 1990s, this service exchanges syringes throughout Vancouver Island. In June 2008 the service was evicted from its fixed Victoria site as a result of a neighbourhood association lawsuit and now operates a mobile needle exchange service in Victoria.

For the present study, eligibility criteria limited participation to persons aged eighteen and over who had injected illicit drugs within the past four months and who were active on the AVI-SOS registry. This registry contains date-specific records of all needle exchanges listed by clients' unique codes. The University of Victoria's Human Research Ethics Board reviewed and approved the survey instrument (Human Subjects' Certificate 08–277). Participants were paid a $20 honorarium for participating in the survey interview, which took less than an hour to administer. The survey included sections pertaining to: 1) basic demographic and educational history, 2) substance use history, 3) current injection practices, 4) egocentric risk networks, 5) worry factors and, 6) drug sharing scenarios.

In total 105 AVI-SOS clientele completed the survey questionnaire. Descriptive statistics for this sample are presented in Tables 1 and 2. These indicate a predominantly male (70%), White (77%), and older (mean age > 40.0 years) sample. Clientele interviewed were characterized by both a long Victorian residency (mean years in Victoria > 17 years) and a lengthy affiliation with the needle exchange (mean time period needle exchange client > 7 years). Despite fairly high levels of formal education and literacy, with almost one-half (48%) completing high school, slightly more than half the interviewees were homeless at the time of the survey (average number of places slept in last week = 2.5) and another 10% were living in shelters. Overall the sample consisted of older, street-entrenched injection drug users with both a long Victoria residence, and a lengthy association with the AVI-SOS needle exchange program.

**Table 1:** AVI-SOS Clientele descriptive statistics for 105 individuals

**Variable**	**N**	**%**
**Gender**		
Male	**74**	**70**
Female	**30**	**29**
Transgender	**1**	**1**
**Ethnicity**		
White	**81**	**77**
Black	**3**	**3**
Hispanic	**0**	**0**
Aboriginal	**15**	**14**
Metis	**6**	**6**
**Education**		
Grades 1–8	**16**	**15**
Attended High School	**39**	**37**
Graduated High School	**26**	**25**
Post-Secondary	**24**	**23**
**Housing Situation**		
Own/Rent	**41**	**39**
Subsidized Housing	**10**	**10**
Homeless	**54**	**51**

**Table 2:** Sample size, mean, standard deviation, and range for AVI-SOS clientele descriptive variables

**Variable**	**N**	**Mean**	**SD**	**Range**
Age	105	41.6	8.5	19–61
Years lived in Victoria	103	17.3	13.4	0–55
Number of places slept last week	105	2.5	2.0	1–7
Years needle exchange client	105	7.2	5.3	0–19

To investigate worry patterns survey participants scored 14 items on a five-point scale measuring how frequently they worry about each item, with the scale being: 1 = Never, 2 = Once a month, 3 = Weekly, 4 = Daily, 5 = All the time. While response rates to sections within the total survey varied, for example over 10% chose not to complete the network questions, all 105 participants completed this section which was written by AVI-SOS staff in collaboration with needle exchange clientele and pretested to ensure that questions representing risks and challenges Victoria PWUID face daily were included, and that question wording and terminology were clear. In the actual questionnaire administration, both individual clients and interviewers had a copy of the questionnaire and went through each section together to ensure mutual understanding of the instrument's questions.

From discussions with AVI staff and needle exchange clientele prior to administering the questionnaire the 14 items in the worry section were thought to represent three areas of risk and worry for PWUID. These were:

**1: Overall Security **– this included worry about food and housing as well as personal safety, denoted on the questionnaire as the following items (item number in parentheses): Having a place to stay (8), Able to get food (10), Being robbed (7), Being assaulted (9), Being farmed (robbed while sleeping or high) by your peers (11) and Police arrest (or being jacked up by police) (2).

**2: Injection Drug Use-Specific Worries **– included here were: Overdosing (1), Vein damage (6), Missing your smash (or vein) (13), Police confiscating syringes (12) and, Getting clean needles (14).

**3: Infectious Disease Worries **– these include worry about contracting HIV/AIDS, (3), Hepatitis C Infection (4) and Sexually Transmitted Infections (5).

To assess whether the survey participants also viewed these multiple items in the same perspective we used the SAS^® ^(Version 9.13) PROC FACTOR sub-routine to perform an exploratory factor analysis on responses to the worry questions. Factor analysis is a data reduction statistical technique designed to delineate a hypothesized underlying structure of large data sets represented by numerous variables [19]. While our data set cannot be considered large, it does meet an important guideline for factor analysis; that the minimum number of subjects in a sample be either 100 subjects or 5 times the number of variables being analyzed, whichever is larger [20:73]. In our sample 105 participants responded to 14 variables, thus qualifying on both criteria.

Factors are assumed responsible for the covariation between two or more observed variables. Based on either correlation or covariance matrices, factor analysis extracts factors representing shared variance. In this paper, factors were extracted using the maximum likelihood option contained within PROC FACTOR, which permits hypothesis testing for the best number of factors to be retained [20]. Extracted factors were then rotated, that is, a linear transformation was performed on the factor solution for easier interpretation, via the PROMAX option in SAS, rendering the extracted factors correlated or "oblique". Individual variables were considered to "strongly load" on each factor if they possessed factor loading scores equal to or above 0.40 [19:29]. Variables which did not achieve this level (known as low-loading) were removed from subsequent analysis.

To determine the number of factors to be retained in the model a scree test or plot depicting each of the variables as a separate factor with respect to its corresponding eigenvalue (interpreted as the amount of variance accounted for by each factor) was constructed. The point at which the slope of the plot changes from a rapid to a slow decline is the cut-off for the number of factors to be retained. This point separates factors with large eigenvalues from those with relatively small eigenvalues [21]. In addition, as maximum likelihood techniques were used to estimate the factor coefficients, a Chi-square test of the hypothesis that k factors are sufficient was performed.

## Results

### Univariate results and reliability estimates

To first measure the degree of worry recorded for each variable and to determine if they are associated, we calculated their means and standard deviations. These results are presented in Table 3 with the individual variables placed according to their proposed factor. As seen here there was a wide range exhibited in the mean values for each variable, ranging from the highest value (mean = 3.11) associated with having a place to stay, to the lowest values recorded for being able to obtain clean needles (mean = 1.72) and worry about police arrest (mean = 1.76). Worry about HIV/AIDS had the fourth highest ranking (mean = 2.84) below only worry about having a place to stay, being robbed (mean = 2.91), and contracting hepatitis C (mean = 2.89).

**Table 3:** Descriptive statistics for individual variables and Cronbach's alpha for each proposed factor

**Overall Security Worries**^1^		
**Variable**	**Mean**	**SD**

Having a Place to Stay	3.11	1.76
Able to Get Food	2.01	1.41
Being Robbed	2.91	1.63
Being Assaulted	2.51	1.56
Being Farmed	2.68	1.66
Being Arrested	2.76	1.59

**Injection Drug Use-Specific Worries**^2^		

**Variable**	**Mean**	**SD**

Overdosing	2.04	1.34
Vein Damage	2.65	1.57
Missing Your Smash	2.56	1.70
Police Confiscating Needles	1.76	1.44
Getting Clean Needles	1.72	0.45

**Infectious Disease Worries**^3^		

**Variable**	**Mean**	**SD**

HIV/AIDS	2.84	1.69
Hepatitis C Infection	2.89	1.71
STIs	2.11	1.50

^1^α = 0.75, ^2^α = 0.71, ^3^α = 0.74

Also shown in Table 3 is a measure of inter-variable reliability known as Cronbach's alpha, which denotes how well the variables in each proposed factor are related. A commonly applied rule of thumb is that alpha levels should equal or exceed 0.70. As shown in Table 3 this level is met for all three of the proposed factors.

### Factor analysis

Factor analysis proceeded in a two-step manner. First, all fourteen variables were included in the analysis, and the results examined for low-loading variables. Two variables, FARMED and ARREST (Questions 3 and 6 respectively) did not load strongly on any factor. Accordingly, in the second step these variables were removed and the analysis repeated. For this second run the corresponding scree plot was constructed. This revealed a steep drop-off in eigenvalues after the first factor, which had an eigenvalue of 5.14. The second factor featured an eigenvalue of 2.02, while the third factor had an eigenvalue of 0.97. Further, there was not enough evidence to reject the hypothesis that 3 factors are sufficient (X^2^_33 _= 33.4, p-value = 0.45). Based on the scree plot and the chi-square test, 3 factors were retained in the model.

Examination of the variable loadings, representing standardized regression coefficients, for each of the three factors is shown in Table 4. These data support our initial supposition of three distinct factors pertaining to: 1) worry about overall personal security, 2) specific worries associated with injection drug use and, 3) worry about contracting infectious diseases associated with injection drug use. The first factor, worry about overall security accounts for over 60% of the total variance and is represented by four manifest variables, being robbed (ROBBED), assaulted (MUGGED), having a place to stay (PLACE) and finding food (FOOD). The second factor contains five variables, worry about overdosing (OD), vein collapse (VEINS), getting clean needles (CLEAN), police taking needles (TAKE) and missing your smash (SMASH), and contributes another 24% of the total variance. The third factor includes the variables relating to worry about contracting HIV/AIDS, hepatitis C (HCV), and sexually transmitted infections (STIs) contributed the remaining 15% of variation.

**Table 4:** Rotated factor pattern and standardized regression coefficients for the 3 factor model.

**Variable**	**Factor 1**	**Factor 2**	**Factor 3**
Overdose	0.00	**0.41**	0.17
HIV	-0.18	0.21	**0.67**
HCV	0.10	-0.14	**0.87**
STI	0.09	0.16	**0.47**
Veins	0.11	**0.42**	0.28
Robbed	**0.70**	-0.01	-0.06
Place	**0.69**	0.01	-0.02
Mugged	**0.76**	-0.03	-0.08
Food	**0.52**	0.08	0.03
Take	0.01	**0.47**	0.08
Smash	0.10	**0.74**	-0.14
Clean	0.08	**0.55**	0.06

Coefficients that loaded on a factor (≥0.40) are in bold

## Discussion

This paper performed an exploratory factor analysis on data pertaining to a broad array of possible worries thought to characterize the daily life of People Who Use Injection Drugs currently enrolled in a needle exchange program administered by AIDS Vancouver Island, in Victoria, British Columbia, Canada. In doing so it was proposed that three common factors representing worry about overall personal security, health concerns specific to injection drug use, and contracting HIV, HCV, and STIs would be delineated. Exploratory factor analysis indicated that the data did indeed contain these three specific factors. Equally important, each factor fulfilled the four interpretability criteria stressed by Hatcher [19:85–86]: 1) at least three variables with significant loadings on each retained factor, 2) variables loading on a given factor share some conceptual meaning, 3) variables loading on different factors appear to measure different constructs and, 4) the rotated factor pattern demonstrates simple structure, i.e. most variables load on only one factor and have near-zero loadings on others. Two variables, worry about being farmed, or robbed while high by one's peers and worry about police arrest, were dropped from the factor analysis, but univariate analysis showed that they both possessed high mean values, and were viewed as additional important risks to our sample.

Our results are limited in being based on a small non-probabilistic sample which hinders generalization to other settings. However, we note that this analysis corresponds to previous studies [13,14] indicating that for PWUID specific worry about HIV/AIDS exists alongside general living and security considerations.

Consideration of all these concerns echoes the classic paper by Strathdee et al. [22] that argued that "needle exchange is not enough", and that while vital, needle exchange programs should be, "... considered one component of a comprehensive programme including counselling, support and education". More than a decade later these words still ring true, with ethnographic [15] and survey – based [16] studies linking social instability (e.g. homelessness), to both heightened HIV risk behaviour and diminished adherence to anti-retroviral treatment therapy.

While certainly not detracting from the large number of rigorous studies indicating multiple positive HIV/AIDS related harm reduction effects associated with needle exchange programs (for a recent listing of these see [1:143]), our results again emphasize the need to address larger structural problems which form risks and worries for PWUID. Unfortunately in the present case the closure of the AVI-SOS fixed site needle exchange facility limits the organization's ability to address these broader programs. Throughout its existence the fixed-site provided a suite of services, ranging from providing hot meals through access to street nurses, referrals to housing/shelter organizations, personal counsellors and HIV/HCV testing to simply providing a safe, dry, warm place. With its closure AVI-SOS must attempt to provide these services via newly established outreach services which cannot individually offer the array of services provided by the now defunct fixed-site; which this analysis reveals their clientele want and need.

In conclusion, combined with the historically more frequently applied concept of risk, consideration and inclusion of PWUID's panoply of everyday worries into broader-based harm reduction interventions could provide important insights or "windows" into their lives and yield effective programs featuring the convergence of PWUID perspectives and public health goals.

## Competing interests

The authors declare that they have no competing interests.

## Authors' contributions

HE, EKG, JL, RS and EAR designed the study questionnaire, constructed the research design, collected the data upon which this analysis is based, and interpreted analysis results. LC, RS and EAR completed the statistical analysis, and wrote the manuscript draft. All authors read and approved the final manuscript.
